# Absence of Susceptibility Vessel Sign in Patients With Malignancy-Related Acute Ischemic Stroke Treated With Mechanical Thrombectomy

**DOI:** 10.3389/fneur.2022.930635

**Published:** 2022-07-14

**Authors:** Morin Beyeler, Nebiyat F. Belachew, Moritz Kielkopf, Enrique B. Aleman, Alejandro Xavier León Betancourt, Kotryna Genceviciute, Christoph Kurmann, Lorenz Grunder, Barbara Birner, Thomas R. Meinel, Adrian Scutelnic, Philipp Bücke, David J. Seiffge, Tomas Dobrocky, Eike I. Piechowiak, Sara Pilgram-Pastor, Heinrich P. Mattle, Pasquale Mordasini, Marcel Arnold, Urs Fischer, Thomas Pabst, Jan Gralla, Martin D. Berger, Simon Jung, Johannes Kaesmacher

**Affiliations:** ^1^Department of Neurology, Inselspital, Bern University Hospital, University of Bern, Bern, Switzerland; ^2^Graduate School for Health Sciences, University of Bern, Bern, Switzerland; ^3^Institute for Diagnostic and Interventional Neuroradiology, Inselspital, Bern University Hospital, University of Bern, Bern, Switzerland; ^4^Department of Neuroradiology, Faculty of Medicine, Medical Center – University of Freiburg, University of Freiburg, Freiburg, Germany; ^5^Department of Neurology, University Hospital of Basel, University of Basel, Basel, Switzerland; ^6^Department of Medical Oncology, Inselspital, Bern University Hospital, University of Bern, Bern, Switzerland

**Keywords:** susceptibility vessel sign, malignancy-related stroke, biomarkers, paraneoplastic coagulation disorders, ischemic stroke, mechanical thrombectomy, thrombus composition/occult malignancy

## Abstract

**Background and Purpose:**

Clots rich in platelets and fibrin retrieved from patients with acute ischemic stroke (AIS) have been shown to be independently associated with the absence of the susceptibility vessel sign (SVS) on MRI and active malignancy. This study analyzed the association of SVS and the presence of active malignancy in patients with AIS who underwent mechanical thrombectomy (MT).

**Methods:**

This single-center, retrospective, and cross-sectional study included consecutive patients with AIS with admission MRI treated with MT between January 2010 and December 2018. SVS status was evaluated on susceptibility-weighted imaging. Adjusted odds ratios (aORs) were calculated to determine the association between absent SVS and the presence of active or occult malignancy. The performance of predictive models incorporating and excluding SVS status was compared using areas under the receiver operating characteristics curve (auROC).

**Results:**

Of 577 patients with AIS with assessable SVS status, 40 (6.9%) had a documented active malignancy and 72 (12.5%) showed no SVS. The absence of SVS was associated with active malignancy (aOR 4.85, 95% CI 1.94–12.11) or occult malignancy (aOR 11.42, 95% CI 2.36–55.20). The auROC of predictive models, including demographics and common malignancy biomarkers, was higher but not significant (0.85 vs. 0.81, *p* = 0.07) when SVS status was included.

**Conclusion:**

Absence of SVS on admission MRI of patients with AIS undergoing MT is associated with malignancy, regardless of whether known or occult. Therefore, the SVS might be helpful in detecting paraneoplastic coagulation disorders and occult malignancy in patients with AIS.

## Introduction

Patients with malignancy-related strokes are more likely to suffer more severe or fatal or recurrent strokes compared to other patients with stroke (1–3). Identifying patients with malignancy in the acute stroke setting would allow targeted treatment and secondary prevention and might improve outcome (4, 5). Thus, easily accessible biomarkers associated with malignancy are needed. Clots in patients with stroke with active malignancy retrieved *via* mechanical thrombectomy (MT) have been shown to contain more fibrin and platelets than clots from other patients (4, 6). A noninvasive, *in situ* characterization of clots may be achieved through appropriate imaging. Whereas computed tomography (CT) merely provides information on the density of the clot, magnetic resonance imaging (MRI) may allow more sophisticated characterization of their composition. The susceptibility vessel sign (SVS) identified on T2^*^ gradient recalled echo imaging (T2^*^ GRE) and susceptibility-weighted imaging (SWI) in brain MRI may serve this purpose (7, 8). The underlying stroke etiology influences the clot composition and, consequently, the SVS status on cerebral imaging (9). Thrombi from cardioembolic stroke and large artery atherosclerosis stroke (LAA) have a high proportion of erythrocytes in their histopathological composition (10, 11). Interestingly, a study also described a high proportion of platelets in thrombi from patients with LAA (12). Currently, only cardioembolic stroke has been associated with the presence of SVS (13, 14). In opposition, clots due to hypercoagulation states (a.o. disseminated intravascular coagulation by underlying malignancy) tend to be composed of more fibrin and platelet and seem to be more associated with absence of SVS (11, 15). Based on the current data, we hypothesized that the absence of SVS, which may indicate platelet and fibrin-rich thrombi, may also be associated with active malignancy. The aim of this study was to test this hypothesis and analyze models that include the SVS to predict the likelihood of active malignancy in patients with AIS.

## Methods

### Study Cohort

This retrospective study evaluated all consecutive patients with stroke treated with MT at our comprehensive stroke center between January 1, 2010, and December 31, 2018. Inclusion criteria were as follows: (1) Ischemic stroke with at least one intracranial symptomatic occlusion on angiography, (2) MRI and SWI performed at admission, (3) SVS status assessable, and (4) MT performed. Patients who received intravenous thrombolytics before blood for laboratory analyses was drawn were excluded from some subanalyses. The local ethics committee approved the study in accordance with Swiss law (reference ID: 2019-00547, Kantonale Ethikkomission Bern). Study approval was restricted to patients treated with MT.

### Definition of Active Malignancy

Active malignancy was defined according to the Haemostasis and Malignancy Scientific and Standardization Committee of the International Society on Thrombosis and Haemostasis (16, 17). Malignancy diagnosed within 1 year after the index stroke was defined as occult malignancy. However, occult malignancy was also considered active at the time of stroke and thus represented a subset of the active malignancy group (18–20). Patients with focal nonmelanoma skin cancer were excluded due to the nonsystemic nature of the disease and its low risk of metastatic spread (21). Patients receiving secondary prophylactic hormone therapy after breast cancer were considered in complete remission and without active malignancy (22, 23).

### Imaging Analysis

**Figure 1:**
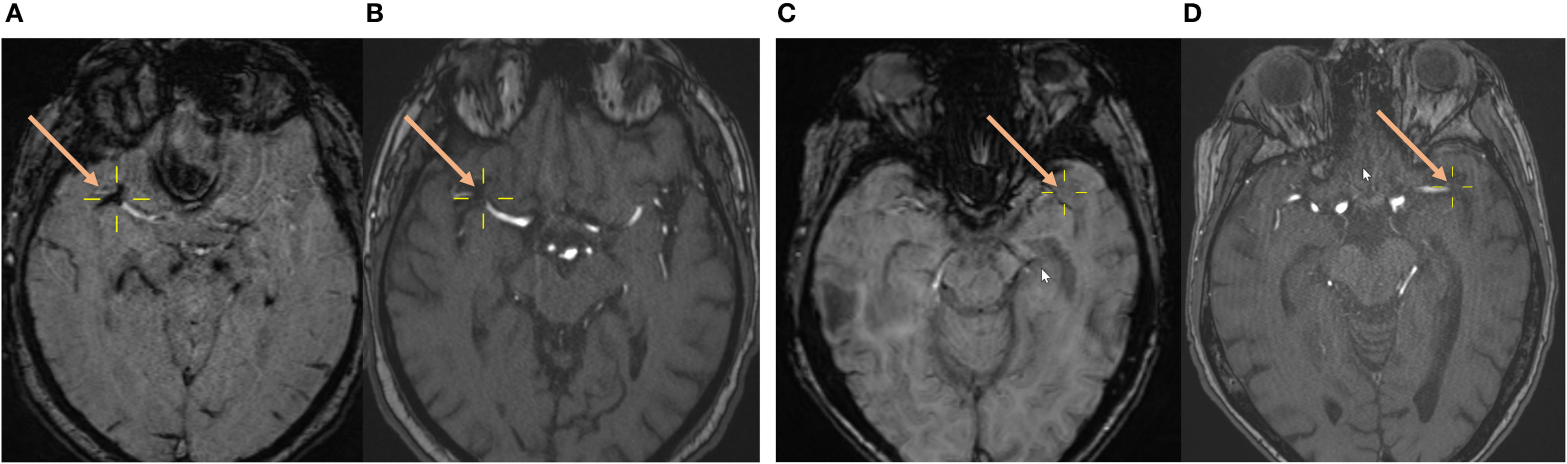
Assessment of the susceptibility vessel sign status on baseline brain MRI. **(A,B)** A 74-year-old male patient with AIS and visible SVS as a circumscribed signal loss on the SWI **(A)** with complete occlusion of the right MCA (M1 segment) on arterial TOF **(B)**. **(C,D)** A 77-year-old female patient with AIS and absent SVS on the SWI **(C)** despite complete occlusion of the left MCA (M1 segment) on arterial TOF **(D)**. *Yellow crosshairs* and *salmon arrows* center, respectively, point to the proximal part of the vessel occlusion on SWI and TOF. AIS, acute ischemic stroke; MCA, middle cerebral artery; SVS, susceptibility vessel sign; SWI, susceptibility-weighted imaging; TOF, time-of-flight angiography.

Imaging was performed on a 1.5 T or 3 T MR imaging scanner (1.5 T: Magnetom Avanto or Magnetom Aera; 3T: Magnetom Verio; Siemens). Magnetom Avanto 1.5 T SWI and 1.5 T Magnetom Aera SWI were performed with the following parameters: TR, 49 ms; TE, 40 ms; flip angle, 15.0°; section thickness, 1.6, 1.8, or 2.0 mm; and intersection gap, 0 mm. Magnetom Verio 3T SWI was performed with the following parameters: TR, 27 ms; TE, 20 ms; flip angle, 15.0°; section thickness, 2.0 mm; and intersection gap, 0 mm. The detailed method used to determine the presence of SVS in the cohort analyzed in this article was described by N. Belachew et al. in a previous publication (24). To summarize, SVS status was assessed retrospectively by two independent neuroradiologists (N.F.B. and E.B.A.). Both raters were blinded to clinical information and outcome, and were not involved in any patient treatment. Regardless of its diameter, SVS was determined to be present if a distinct signal loss corresponding to an occluded and symptomatic intracranial artery could be identified (Figures 1A,B), for which there was no alternative explanation (i.e., neighboring vein, petechial hemorrhage, or microcalcification in the neighboring parenchyma). If no such signal loss could be identified even though a symptomatic vessel occlusion was seen on angiography, SVS was determined to be absent (Figures 1C,D). Interrater reliability regarding SVS evaluation for the study cohort analyzed in this article has been assessed and published in a previous study showing very good correlation (Cohen's κ = 0.873, *p* < 0.001) (24).

### Data Collection

Demographics and baseline stroke characteristics were extracted from the local stroke registry. They included gender, age at admission, prestroke independence (defined as a modified Rankin Scale ≤ 2), blood pressure at admission, prior anticoagulation/antiplatelet therapy, prior statin therapy, cardiovascular risk factors (such as hypertension, diabetes, hyperlipidemia, smoking), National Institutes of Health Stroke Scale on admission, time between symptom-onset/last-seen-well and admission, intravenous thrombolytics before MT, and site of occlusion. Two neurology fellows (M.B. and M.K.), both blinded to the SVS status, retrospectively identified active malignancy (known or occult at the time of stroke) from histological and clinical reports present in the clinical information system. After patients' identification, malignancy localization, histological type, metastatic state, and time metrics related to the malignancy diagnosis and treatment were extracted. The presence of multiterritory infarcts and the assigned stroke etiology at discharge, defined by the Trial of ORG 10172 in Acute Stroke Treatment (TOAST) classification, was also extracted from neuroradiological reports and discharge letters (25). According to the TOAST classification, strokes associated with patent foramen ovale were classified as cardioembolic stroke. A subdivision between intracranial atherosclerosis and artery-to-artery embolism was made for LAA. Embolic stroke of undetermined source (ESUS) was assumed when nonlacunar ischemic stroke occurred in patients in which another underlying stroke etiology in line with the definition of the “NAVIGATE ESUS” trial could not be determined (26). The following laboratory values at admission were extracted from the hospital's clinical information system: D-dimer, fibrinogen, hemoglobin (Hb), C-reactive protein (CRP), leukocytes, thrombocytes, international normalized ratio (INR), thrombin time, and activated partial thromboplastin clotting time.

### Statistical Analysis

Baseline characteristics were compared using Fisher's exact test for categorical variables and Mann–Whitney *U-*test for continuous variables. Descriptive statistics were reported as number and percentage for categorical variables, and median and interquartile range (25–75%) for continuous variables. A logistic transformation was applied if the distribution of continuous values was skewed. The association between the SVS status and active malignancy was assessed using simple and multivariable logistic regression models. Results are displayed as odds ratios (ORs) and adjusted odds ratios (aORs). The association of SVS status with occult malignancies was assessed in a sensitivity analysis. All models were adjusted for demographic characteristics (such as gender and age at admission) and malignancy biomarkers known to be associated with malignancy-related stroke such as ESUS, multiterritory infarcts, D-dimer, leukocytes, CRP, INR, and Hb. The predictive value of SVS status was evaluated by assessing sensitivity, specificity, positive predictive value (PPV), negative predictive value (NPV), positive likelihood ratio (LR+), and negative likelihood ratio (LR–). The performance of predictive models, including and excluding SVS status, was assessed by calculating areas under the receiver operating characteristics curve (auROC). The auROCs were cross-validated using bootstrapping, whereas they were compared using the DeLong test. All statistical analyses were performed using the Stata 16 and R software (version 3.6.0, R Core Team). The current STROBE (Strengthening the Reporting of Observational Studies in Epidemiology) checklist for cross-sectional studies was used to report this study.

## Results

### Study Population

Between January 2010 and December 2018, 1,317 patients with AIS were treated with MT at our stroke center. As already described by Belachew et al. and shown in Supplementary Figure I (study flowchart), 577 patients had SWI with assessable SVS status available at admission and met the inclusion criteria (24). Evidence of active malignancy at the time of stroke was found in 40 patients (6.93%), of which a subset of 9 patients (1.56%) had occult malignancy. Active malignancy characteristics are summarized in Supplementary Figure II. A total of eight patients (1.39%) were excluded from subgroup analysis of blood markers as they had received intravenous thrombolytics before blood was drawn for further analysis.

### Baseline Characteristics

The characteristics of patients with and without active malignancy are summarized in Supplementary Table I. SVS was absent in 12.5% of all patients (*n* = 72/577). Of the patients with active malignancy, SVS was absent in 37.5% (*n* = 15/40), while only 4.3% (57/537, *p* < 0.001) of patients without active malignancy lacked the SVS. Patients with active malignancy were more likely to be functionally dependent before the index stroke, had lower diastolic blood pressure (<90 mmHg), and more often showed anterior and more distal middle cerebral artery occlusions. Intravenous thrombolysis before MT was less often administered to patients with stroke with active malignancy compared to patients without malignancy. ESUS and multiterritory infarcts were more frequent in the group with active malignancy. Active malignancy was associated with elevated D-dimer, low Hb, elevated CRP, elevated leukocytes, and higher INR.

### Association of Active Malignancy With Absence of SVS and Other Biomarkers

**Figure 2:**
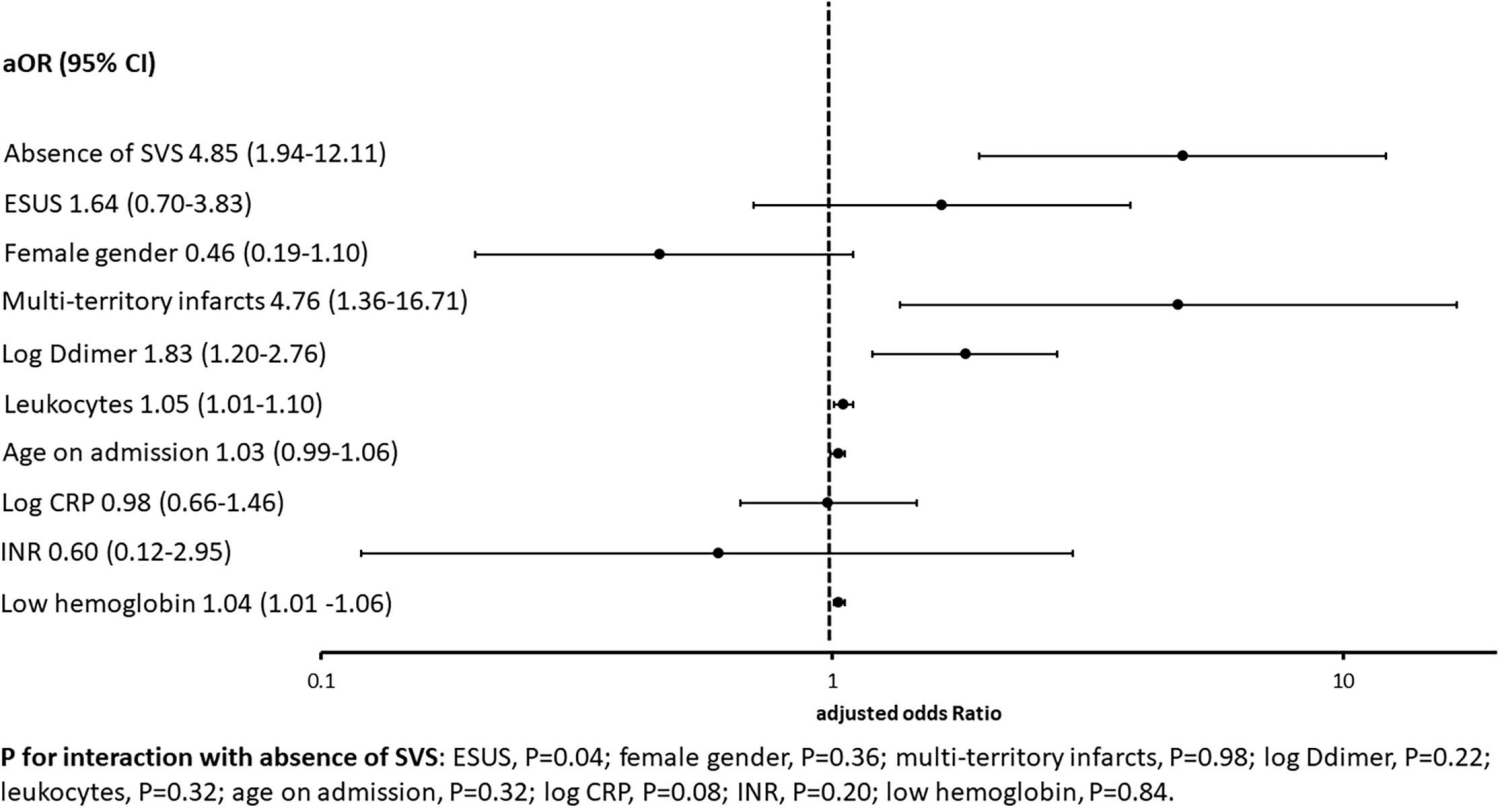
Association between active malignancy, neuroimaging, and blood biomarkers in the multivariable logistic regression. According to the primary goal of this study and previous evidence, adjusted odds ratios (aOR) and their 95% confidence intervals (95% CI) for the association between active malignancy and preselected biomarkers are summarized in this figure. Absence of SVS showed the strongest association with the presence of active malignancy, followed by multiterritory infarcts. CRP, C-reactive protein; ESUS, embolic stroke of undetermined source; INR, international normalized ratio; SVS, susceptibility vessel sign.

Simple logistic regression demonstrated absent SVS to be associated with active malignancy, multiterritory infarcts, elevated D-dimer, ESUS, elevated CRP, elevated leukocytes, and low Hb. All simple regression analyses are summarized in Supplementary Figure III. Multivariable regression analyses showed significant associations of active malignancy and absent SVS, multiterritory infarcts, elevated D-dimer, elevated leukocytes, and low Hb. Results of multivariable regression analyses, including ORs and 95% confidence intervals, are summarized in Figure 2.

### Diagnostic Value of SVS Status in Identifying Active Malignancy

**Figure 3:**
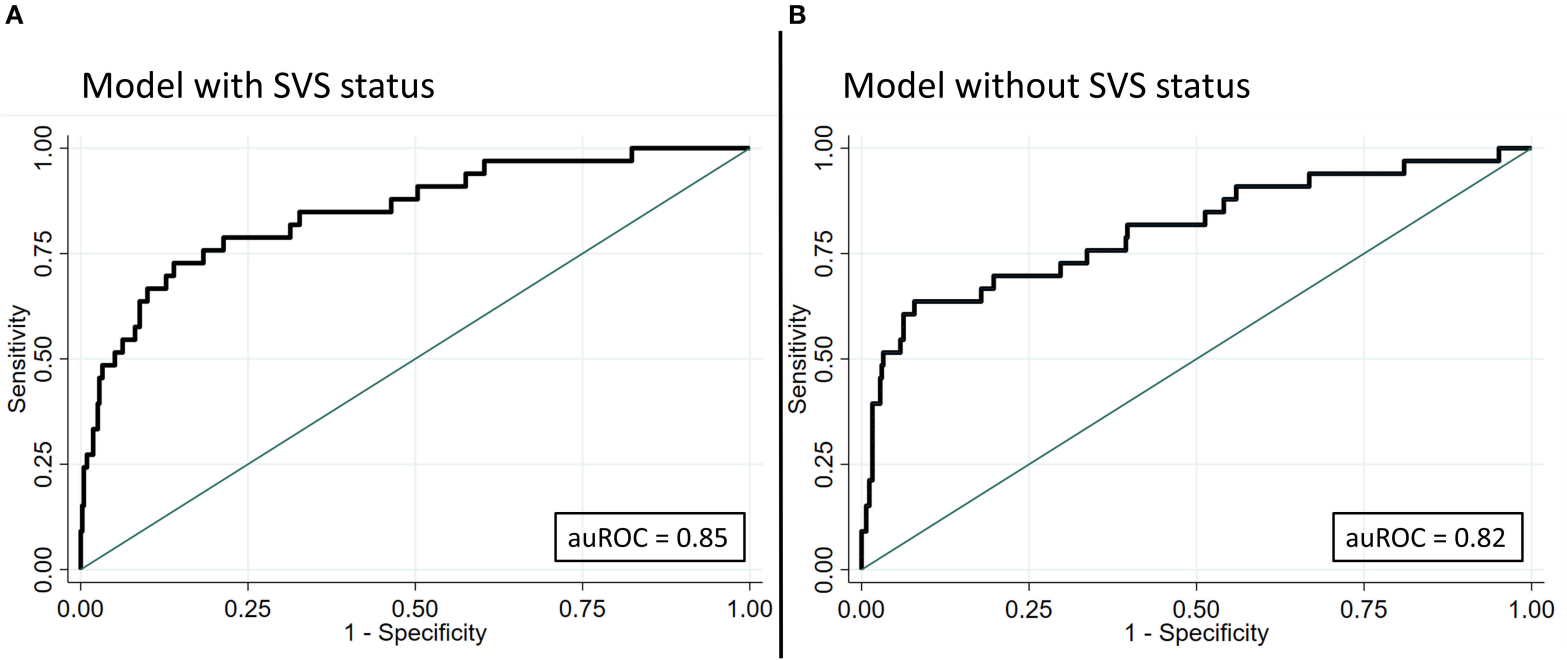
Comparison of predictive models for active malignancy with and without SVS status. The covariables used for the predictive models were age at admission and gender and variables associated with active malignancy in the multivariable logistic regression (ESUS, multiterritory infarcts, D-dimer, Hb, leukocytes). The auROC of the model with SVS status was 0.85 (95% CI 0.78–0.92, **A**). The internal cross-validation demonstrated no variation of the auROC and 95% CI. In the absence of the SVS status, the auROC of the model was 0.81 (95% CI 0.72–0.90, **B**), the internal cross-validation showed an auROC of 0.82 (95% CI 0.68–0.90). The DeLong test did not show a significant difference between the two models (*p* = 0.074). auROC, area under the receiver operating characteristics curve; CRP, C-reactive protein; ESUS, embolic stroke of undetermined source; Hb, hemoglobin; SVS, susceptibility vessel sign.

Absence of SVS alone predicted active malignancy as follows: sensitivity 20.83%, specificity 95.05%, PPV 37.5%, NPV 89.38%, LR+ 4.21, and LR– 0.83. Predictive models incorporating age at admission, gender, and variables associated with active malignancy in the multivariate logistic regression as well as SVS status were tested. The auROC of the model, including SVS status, was 0.85 (95% CI 0.78–0.92, Figure 3A). The internal cross-validation using bootstrapping demonstrated no variation of auROC and 95% CI. If SVS status was excluded, the auROC of the model was 0.81 (95% CI 0.72–0.90, Figure 3B) while the internal cross-validation showed an auROC of 0.82 (95% CI 0.68–0.90). The DeLong test did not find a significant difference between the two models (*p* = 0.07).

Subgroup analyses using the same covariates as in the multivariate logistic regression showed that the absence of SVS remained associated with occult malignancy alone (aOR 11.42, 95% CI 2.36–55.20, *p* = 0.002) when patients with active malignancy diagnosed before the stroke were excluded. Absence of SVS predicted occult malignancy as follows: sensitivity 8.06%, specificity 99.17%, PPV 55.50%, NPV 89.38%, LR+ 9.76, and LR– 0.93.

### Subgroup Analyses According to the Underlying Stroke Etiology

According to the assigned stroke etiology, the point estimate of the association between absence of SVS and the presence of active malignancy differed slightly (Supplementary Figure IV). However, there was no significant heterogeneity of the association between absence of SVS and active malignancy (*p* = 0.24). After excluding patients with LAA (*n* = 74, two without SVS), the aOR for the association decreased from 4.89 (95% CI 1.94–12.11, Figure 2) to aOR 3.9 (95% CI 1.42–10.50). Excluding only cases where intracranial stenosis was the stroke etiology (*n* = 11, one without SVS) the aOR decreased to 4.77 (95% CI 1.91–11.89).

## Discussion

The main findings of this study are as follows: (1) absence of SVS is associated with both active and occult malignancy in patients with ischemic stroke treated with MT; (2) absence of SVS in admission MRI as imaging biomarker may increase the performance of predictive models for active malignancy in patients with AIS; and (3) the association between the absence of SVS and active malignancy seems not to be influenced by the assigned etiology at discharge. Approximately 8% of patients with acute stroke have a concomitant active malignancy (known or occult) at the time of stroke (4, 27). Identifying the malignancy as the potential cause of the stroke in these patients is essential to guide secondary prevention, which includes therapeutic anticoagulation with low-molecular-weight heparin (4, 5). Strokes with concomitant malignancy are associated with nonspecific biomarkers such as elevated D-dimer, elevated CRP, elevated fibrinogen, elevated lactate dehydrogenase, low Hb, undetermined stroke etiology (especially ESUS), and multiterritorial infarcts in brain imaging (3, 4, 18, 28). These biomarkers may help to detect a malignancy-related coagulation disorder. However, proving the causality between active malignancy and an ischemic stroke event remains difficult. Considering the potential clinical ramifications, the search for further biomarkers that may improve the likelihood of detecting malignancy-related coagulopathy and occult malignancy at the time of stroke is warranted. Finelli et al. were the first to describe multiterritory infarcts seen on diffusion-weighted imaging as an independent imaging biomarker for malignancy-related stroke (29). Guo et al. demonstrated a specificity of 0.65 and a sensitivity of 0.92 for the prediction of occult malignancy in patients with stroke with undetermined stroke etiology if two or more vascular territories are affected (30). The results of our study add to the current evidence regarding the advantage of brain MRI in patients with malignancy-related stroke.

Susceptibility-weighted sequences such as T2^*^ GRE and SWI are central to the MRI-based acute stroke workup (31, 32). SWI sequences provide reliable information on a potential hemorrhagic transformation but may also outline the occluded vessel depending on clot composition (9, 13, 15, 33, 34). Among others, Zhang et al. reported SVS to be present in 90% of cardioembolic strokes (*n* = 35/39), 53.5% of large-artery atherosclerosis strokes (*n* = 23/43), and 75.9% of strokes with undetermined etiology (*n* = 22/29), indicating that SVS status may differ depending on stroke etiology (13, 33, 34). However, a recent meta-analysis confirmed only the association between SVS and cardioembolic stroke (14). Histopathological analyses after MT have shown that thrombi with a cardioembolic genesis predominantly contain erythrocytes (35). Platelet and fibrin-rich thrombi have been shown to be independently associated with malignancy-related stroke, hypercoagulation, and the absence of SVS (4, 6, 11, 15, 36). Our study adds to this knowledge. It demonstrates that absent SVS is associated with active malignancy in AIS treated with MT, irrespective of whether known or unknown at time of stroke. Although the association between the absence of SVS and the presence of malignancy differed slightly by the assigned stroke etiology (Supplementary Figure IV), the lack of interaction did not allow any conclusions regarding the role of the assigned etiology on the relation between the absence of SVS and the presence of active malignancy. The exclusion of LAA did not increase the association between the absence of SVS and malignancy in our study population, thus suggesting no association between absent SVS and LAA (13). Predictive models for diagnostic workup are generally developed to help in decision-making for further investigations in complex clinical situations (37, 38). However, this study did not aim to conceptualize a new predictive model for active malignancy but rather to validate the SVS status as an additional biomarker. Although the difference in the auROCs only tended to be greater with SVS included, a more sophisticated SVS evaluation (i.e., quantitative susceptibility mapping) may increase predictive performance (39, 40). As MRI may not always be available or practicable in the acute stroke setting, density, which has also been shown to correlate with erythrocyte content, may be used in patients imaged with CT (33). Furthermore, the macroscopic characteristics of clots retrieved with MT (“white” indicating fibrin-rich thrombi vs. “red-black” indicating erythrocyte-rich thrombi) may provide additional hints about their composition (36, 41). In the same context, Bourcier et al. recently demonstrated an association between “red-black” clots and the presence of SVS, strengthening the evidence that SVS status is a reliable biomarker for their composition (15).

## Limitations

This study has several limitations. First, this was a monocentric and retrospective study, which may limit generalizability of results. Second, in accordance with the terms of the ethical approval, we included only patients who underwent MT. Further studies are needed to confirm our findings in a more general stroke population. Third, although the percentage of malignancies is consistent with studies in the literature, the small number of active or occult malignancies may reduce the statistical power of the results obtained. Additionally, the small number of malignancies precluded accurate subgroups analyses regarding assigned stroke etiology. Because the underlying etiology may influence the thrombus composition, further studies with more patients are needed. Fourth, the high rate of SVS reported in this study may result from a selection bias due to a specific subpopulation of patients with stroke eligible for thrombectomy and the inhomogeneous use of 1.5 and 3 T MRI. Fifth, concomitant microscopic analysis of the retrieved thrombi would have provided a more robust validation of the study hypothesis, assuming that the absence of SVS is a surrogate marker of platelet and fibrin-rich thrombi in case of malignancy-related stroke. Sixth, the comparison of predictive models is limited by the lack of an external validation group and by power considerations comparing auROC of different models according to the number of active malignancies. Finally, levels of lactate dehydrogenase, a well-known marker for the presence of active malignancy, were not available (3).

## Conclusion

Absent SVS on baseline MRI is associated with active malignancy in patients with AIS undergoing MT, regardless of whether the malignancy is known or unknown at the time of stroke. Using SVS as surrogate marker for clot composition may increase the chances of detecting occult malignancy in patients with AIS, which could be helpful both for the treatment of the malignancy and secondary stroke prevention and thus may lead to better outcomes.

## Data Availability Statement

Data are available upon reasonable request, access requests should be directed to the authors.

## Ethics Statement

The studies involving human participants were reviewed and approved by Kantonale Ethikkomission Bern. Written informed consent for participation was not required for this study in accordance with the national legislation and the institutional requirements.

## Author Contributions

MB contributed to conception and design, data acquisition, analysis and interpretation of data, and writing of the publication. NB contributed to conception and design, data acquisition and writing of the publication. MK and EA contributed to data acquisition and critical revision of the manuscript for important intellectual content. KG and BB contributed to conception and design, and critical revision of the manuscript for important intellectual content. SJ and JK contributed to conception and design, critical revision of the publication for important intellectual content, and supervision. AL, CK, LG, TM, AS, PB, DS, TD, EP, SP-P, HM, PM, MA, UF, TP, JG, and MDB contributed to interpretation of data and critical revision of the manuscript for important intellectual content. All authors contributed to the article and approved the submitted version.

## Conflict of Interest

TM reports research support from the Bangerter Rhyner Foundation, Swiss National Foundation, and the Swiss Heart Foundation. HM reports personal consulting fees outside of this study from Servier, Bayer, Medtronic, Stryker and Cerenovus. PM reports receipt of research support from Siemens, Cerenovus, iSchmaview, Medtronic, Stryker, the Swiss Heart Foundation and the Swiss National Foundation, receipt of consultant fees paid to the institution from Medtronic, Cerenovus, Phenox and Microvention during the conduct of the study, unrelated to the submitted work. MA reports personal fees from Bayer, Bristol-Myers Squibb, Medtronic, Amgen, Daiichi Sankyo, Nestlé Health Sciences, Boehringer Ingelheim, and Covidien during the conduct of the study. UF reports grants during the conduct of the study from Medtronic, Stryker, and CSL Behring, unrelated to the submitted work. JK reports grants from the Swiss Academy of Medical Sciences/Bangerter Foundation, Swiss Stroke Society, and Clinical Trials Unit Bern during the conduct of the study. SJ reports grants from the Swiss National Science Foundation and the Swiss Heart Foundation. The remaining authors declare that the research was conducted in the absence of any commercial or financial relationships that could be construed as a potential conflict of interest.

## Publisher's Note

All claims expressed in this article are solely those of the authors and do not necessarily represent those of their affiliated organizations, or those of the publisher, the editors and the reviewers. Any product that may be evaluated in this article, or claim that may be made by its manufacturer, is not guaranteed or endorsed by the publisher.

## Supplementary Material

The Supplementary Material and the STROBE checklist for this article can be found online at: https://www.frontiersin.org/articles/10.3389/fneur.2022.930635/full#supplementary-material

## Abbreviations

AIS: acute ischemic stroke
CRP: C-reactive protein
DWI: diffusion-weighted imaging
ESUS: embolic stroke of undetermined source
INR: international normalized ratio
LAA: large artery atherosclerosis stroke
LR: likelihood ratio
MT: mechanical thrombectomy
NPV: negative predictive value
PPV: positive predictive value
SVS: susceptibility vessel sign
SWI: susceptibility-weighted imaging.
