# Supplementary material for: Absence of Susceptibility Vessel Sign in Patients With Malignancy-Related Acute Ischemic Stroke Treated With Mechanical Thrombectomy
Source: Front Neurol. 2022 Jul 14;13:930635. doi: 10.3389/fneur.2022.930635 (PMC9331190; doi:10.3389/fneur.2022.930635)
Supplement: Supplementary file 1 [file Data_Sheet_1.PDF]

SUPPLEMENTAL MATERIALS

Supplementary Figures

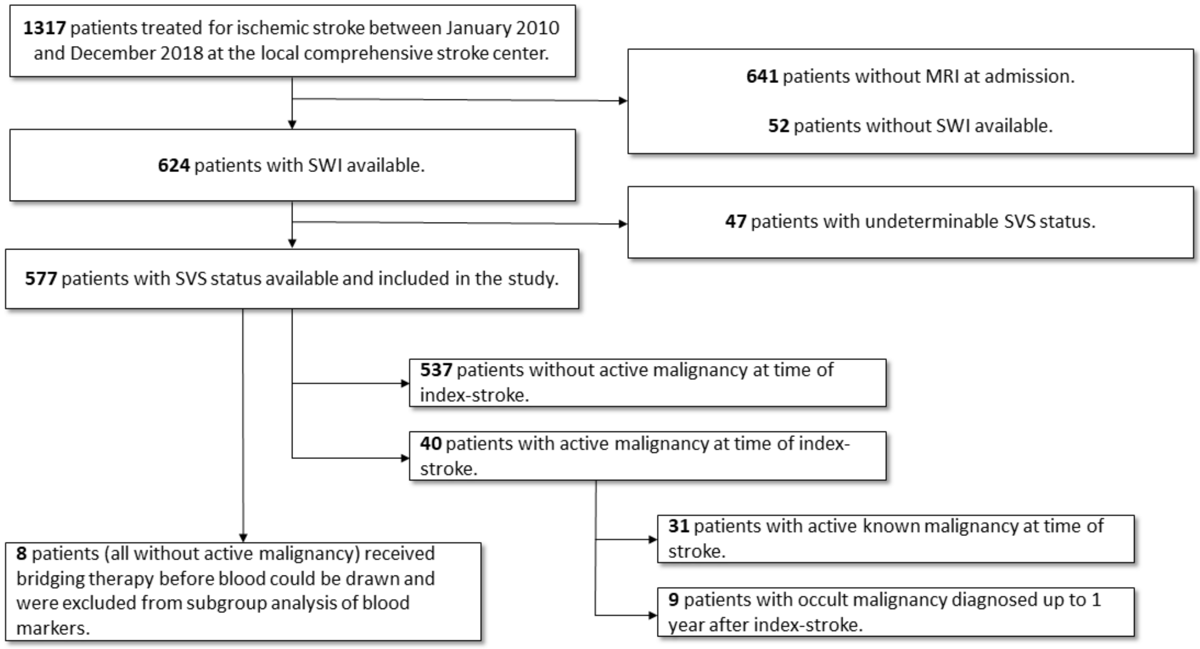

eFigure I – Study flowchart.

This figure shows the process for the inclusion and exclusion of patients in the study. IVT indicates Intravenous thrombolysis; MRI, magnetic resonance imaging; SVS, susceptibility vessel sign; and SWI susceptibility-weighted imaging.

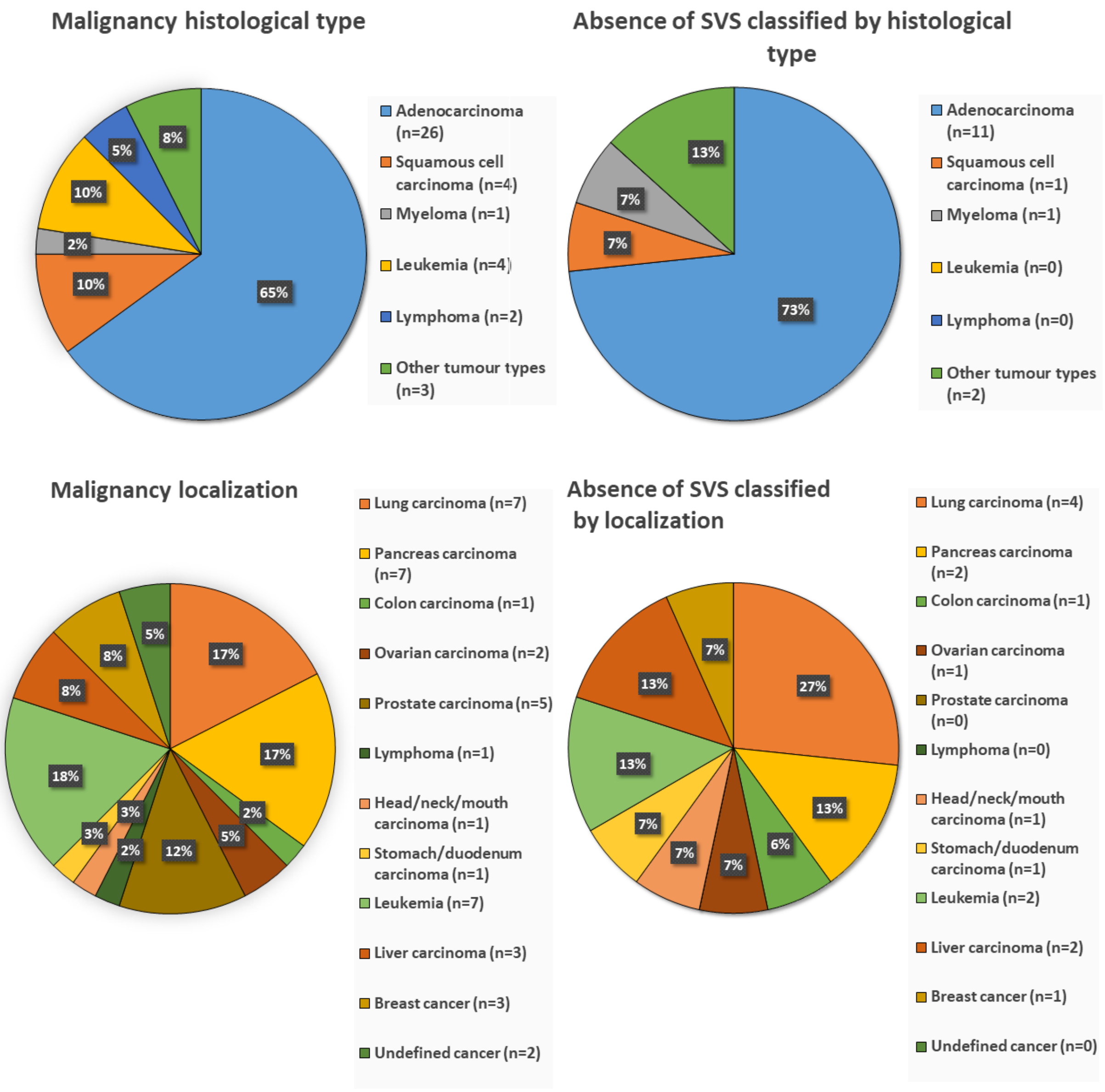

eFigure II – Distribution of malignancy histological types and localizations and relationship with absence of the susceptibility vessel sign (SVS).

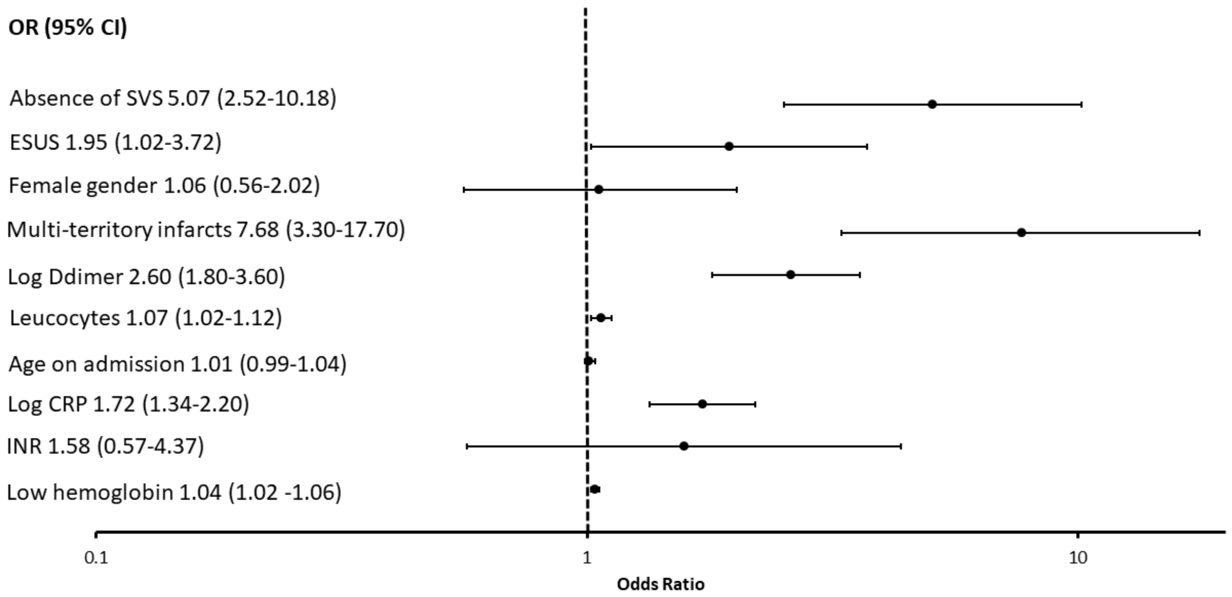

eFigure III – Correlation between active malignancy, and neuro-imaging and blood biomarkers in the simple logistic regression.

According to the goal of this study and previous evidence, odds ratios (OR and their 95% confidence intervals (95% CI) for the association between active malignancy and preselected biomarkers are summarized in this figure. Neuroimaging biomarkers (absence of SVS and multi-territory infarcts) showed the strongest association with the presence of active malignancy. CRP indicates C-reactive protein; ESUS, embolic stroke of undetermined source; INR, international normalized ratio; and SVS, susceptibility vessel sign.

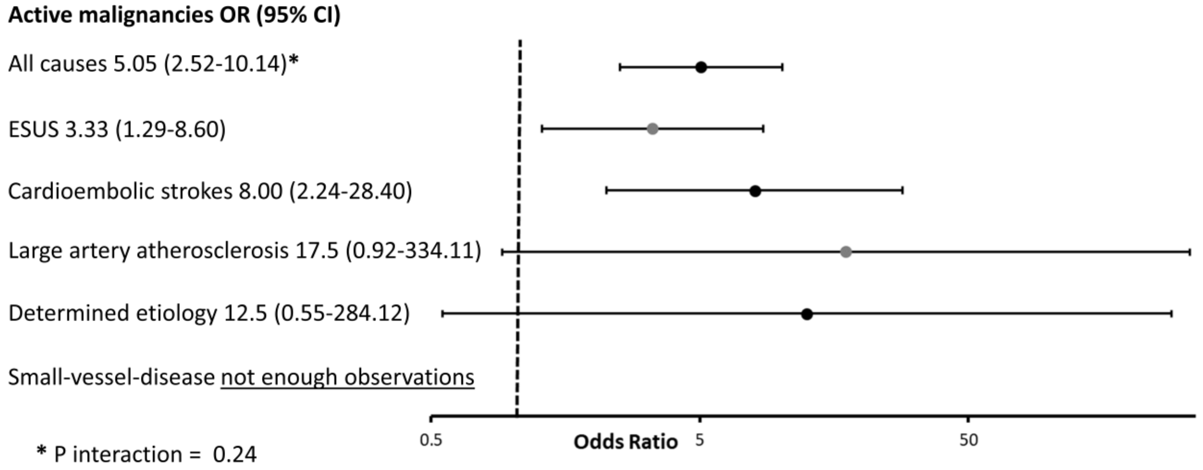

eFigure IV – Association between absence of SVS and active malignancy in subgroup analysis of assigned stroke etiology at discharge. According to the assigned stroke etiology, the point estimate of the association between absence of SVS and the presence of active malignancy differed slightly. However, there was no significant heterogeneity of the association between absence of SVS and active malignancy (P= 0.24). ESUS indicates embolic stroke of undetermined source; OR, odds ratio; SVS, susceptibility vessel sign; 95% CI, 95% confidence interval.

eTable I – Comparison of Baseline Characteristics Between Patients with Active Malignancy Versus no Malignancy.

	All patients (N=577)	No malignancy (N=537)	Active malignancy (N=40)	p-value
Baseline				
Gender, female No. / total No. (%)	296/577 (51.3)	275/537 (51.2)	21/40 (52.5)	1.00
Age at admission (median, IQR)	72.8 (60.8–80.9)	72.9 (60.4–80.9)	72.2 (61.8–79.5)	0.67
Prestroke independence (mRS≤2), No. / total No. (%)	528/576 (91.7)	495/536 (92.4)	33/40 (82.5)	0.039
Systolic blood pressure at admission (median, IQR)	155 (134–174)	154.5 (134–174)	160 (135–175)	0.70
Diastolic blood pressure at admission (median, IQR)	81 (71–95)	82 (72–95)	72 (64–81)	<0.001
Anticoagulation (vitamin K-antagonist and NOAC) prior to stroke, No. / total No. (%)	66/575 (11.5)	535/52 (9.7)	40/14 (35.0)	<0.001
Antiplatelet drugs prior to stroke, No. / total No. (%)	186/575 (32.4)	169/535 (31.6)	17/40 (42.5)	0.16
Prestatin, No. / total No. (%)	148/574 (25.8)	138/534 (25.8)	10/40 (25.0)	1.00
Risk factors				
Previous stroke, No. / total No. (%)	65/577 (11.3)	59/537 (11.0)	6/40 (15.0)	0.44
Hypertension, No. / total No. (%)	378/577 (65.5)	349/537 (65.0)	29/40 (72.5)	0.39
Diabetes, No. / total No. (%)	83/577 (14.4)	77/537 (14.3)	6/40 (15.0)	0.82
Hyperlipidemia, No. / total No. (%)	333/575 (57.9)	309/535 (57.8)	24/40 (60.0)	0.87
Smoking (history or current), No. / total No. (%)	147/576 (25.5)	133/536 (24.8)	14/40 (35.0)	0.19
Coronary artery disease, No. / total No. (%)	89/573 (15.5)	85/535 (15.9)	4/38 (10.5)	0.49
Stroke characteristics				
NIHSS on admission, (median, IQR)	12 (7–17)	12 (7–18)	9 (7–16)	0.19
Time from symptom-onset/last-seen-well to admission in min. (median, IQR)	127 (72–286)	130 (74–285)	92 (60–321)	0.26
IVT prior to MT, No. / total No. (%):	225/577 (39)	220/537 (41.0)	5/40 (12.5)	<0.001
DWI-ASPECTS score (median, IQR)	9 (7–10)	9 (7–10)	8 (8–10)	0.56
**Site of occlusion,** No. / total No. (%):				
Intracranial internal carotid artery	98/577 (17)	94/537 (17.5)	4/40 (10.0)	0.023

Middle cerebral artery (M1)	304/577 (52.7)	284/537 (52.9)	20/40 (50.0)	
Middle cerebral artery (M2)	120/577 (20.8)	105/537 (19.6)	15/40 (37.5)	
Posterior circulation	46/577 (8)	46/537 (8.6)	0/40 (0.0)	
Other occlusions	9/577 (1.6)	8/537 (1.5)	1/40 (2.5)	
**Stroke etiology (TOAST)** No. / total No. (%):				
Cardioembolic	233/577 (40.4)	222/537 (41.3)	11/40 (27.5)	0.41
Small-vessel occlusion	1/577 (0.2)	1/537 (0.2)	0/40 (0.0)	
More than one cause	1/577 (0.2)	1/537 (0.2)	0/40 (0.0)	
Large-artery atherosclerosis	76/577 (13.2)	71/537 (13.2)	5/40 (12.5)	
Stroke of other determined etiology	29/577 (5)	27/537 (5)	2/40 (5.1)	
Stroke of undetermined etiology	237/577 (41.1)	215/537 (40)	22/40 (53.8)	
ESUS, No. / total No. (%)	227/577 (39.3)	205/537 (38.2)	22/40 (55)	0.044
Multi-territory infarcts, No. / total No. (%)	33/577 (5.7)	23/537 (4.3)	10/40 (25.0)	<0.001
Absence of susceptibility vessel sign, No. / total No. (%)	72/577 (12.5)	57/537 (4.3)	15/40 (37.5)	<0.001
Baseline laboratory findings				
Glucose in mmol/L (median, IQR)	6.5 (5.8–7.6)	6.5 (5.8–7.6)	6.8 (6–7.9)	0.25
D-dimer in µg/L (median, IQR)	899 (497–1757)	855 (482–1698)	2505 (909–7896)	<0.001
Hemoglobin in g/L (median, IQR)	135 (124–146)	136 (125–148)	122 (107.5–134)	<0.001
C-reactive protein in mg/L (median, IQR)	3 (3–8)	3 (3–7)	9 (3–24.5)	<0.001
leukocytes in G/L (median, IQR)	8.3 (6.6–10.4)	8.3 (6.6–10.3)	9.5 (6.9–15.9)	0.009
Thrombocytes in G/L (median, IQR)	221 (180–269)	220 (182–268)	234 (167–316)	0.52
Fibrinogen in g/L (median, IQR)	3.1 (2.6–3.7)	3.1 (2.6–3.7)	2.9 (2.2–3.9)	0.33
Thrombin time in sec (median, IQR)	15 (12.9–16.3)	15 (12.9–16.4)	14.7 (14.1–15.6)	0.70
INR (median, IQR)	1 (1–1.1)	1 (1–1.1)	1.1 (1–1.2)	0.009
aPTT (median, IQR)	27.8 (25.4–30.5)	27.9 (25.5–30.5)	26.4 (25–30.7)	0.33

aPTT indicates activated partial thromboplastin time; DWI-ASPECTS, Diffusion-Weighted Imaging-Alberta Stroke Program Early Computed Tomography Scores; ESUS, embolic stroke of undetermined source; INR, International Normalized Ratio; IQR, interquartile range; IVT, Intravenous thrombolysis; MCA, middle cerebral artery; NIHSS, National Institutes of Health Stroke Scale; NOAC, non-vitamin K antagonist oral anticoagulant; TOAST, Trial of ORG 10172 in Acute Stroke Treatment.
